# Treatment‐planning approaches to intensity modulated proton therapy and the impact on dose‐weighted linear energy transfer

**DOI:** 10.1002/acm2.13782

**Published:** 2022-09-26

**Authors:** Austin M. Faught, Lydia J. Wilson, Melissa Gargone, Fakhriddin Pirlepesov, Vadim P. Moskvin, Chia‐Ho Hua

**Affiliations:** ^1^ Department of Radiation Oncology St. Jude Children's Research Hospital Memphis Tennessee USA

**Keywords:** intensity‐modulated proton therapy, linear energy transfer, proton therapy, relative biological effectiveness, treatment planning

## Abstract

**Purpose:**

We quantified the effect of various forward‐based treatment‐planning strategies in proton therapy on dose‐weighted linear energy transfer (LETd). By maintaining the dosimetric quality at a clinically acceptable level, we aimed to evaluate the differences in LETd among various treatment‐planning approaches and their practicality in minimizing biologic uncertainties associated with LETd.

**Method:**

Eight treatment‐planning strategies that are achievable in commercial treatment‐planning systems were applied on a cylindrical water phantom and four pediatric brain tumor cases. Each planning strategy was compared to either an opposed lateral plan (phantom study) or original clinical plan (patient study). Deviations in mean and maximum LETd from clinically acceptable dose distributions were compared.

**Results:**

In the phantom study, using a range shifter and altering the robust scenarios during optimization had the largest effect on the mean clinical target volume LETd, which was reduced from 4.5 to 3.9 keV/μm in both cases. Variations in the intersection angle between beams had the largest effect on LETd in a ring defined 3 to 5 mm outside the target. When beam intersection angles were reduced from opposed laterals (180°) to 120°, 90°, and 60°, corresponding maximum LETd increased from 7.9 to 8.9, 10.9, and 12.2 keV/μm, respectively. A clear trend in mean and maximum LETd variations in the clinical cases could not be established, though spatial distribution of LETd suggested a strong dependence on patient anatomy and treatment geometry.

**Conclusion:**

Changes in LETd from treatment‐plan setup follow intuitive trends in a controlled phantom experiment. Anatomical and other patient‐specific considerations, however, can preclude generalizable strategies in clinical cases. For pediatric cranial radiation therapy, we recommend using opposed lateral treatment fields to treat midline targets.

## 1 INTRODUCTION

The biologic impact of increasing ionization density, quantified by the linear energy transfer (LET), at the distal edge of a Bragg peak is an active area of research in proton radiation therapy.^1^, ^2^, ^3^, ^4^, ^5^, ^6^, ^7^, ^8^, ^9^ Although the association between LET and effective cell killing is clear in vitro, it is not fully understood or described in patients. Direct modeling based on dose‐weighted LET (LETd), two‐parameter modeling accounting for dose and LETd, and phenomenological relative biological effectiveness (RBE) models^5^, ^6^, ^10^, ^11^, ^12^ have had limited success in describing symptomatic effects attributable to radiation.

The urgency to develop a better understanding of how LETd might influence the RBE has been most present when considering pediatric proton therapy patients.^1^ The reduced integral dose and normal tissue sparing in a population potentially more radiosensitive and with decades of life ahead with successful treatment was an early argument for the adoption of proton therapy. As a result, the presence of toxicities that dramatically affect quality of life, although rare, has been an area of intense focus.^5^, ^6^, ^13^, ^14^, ^15^, ^16^ As a department focused on treating pediatric malignancies, a better understanding of how LETd might affect treatment outcomes and how we might create treatment plans with that relationship in mind are things that we must confront daily. The need for deeper understanding and improved tools was highlighted in a recent road map for proton therapy.^17^

Even without evidence‐based biological metrics, means of influencing LETd distributions during the planning process are continually developed and explored. Full inverse optimization of biologically effective dose has been implemented by using in‐house developed systems as reported by Tseung et al.^18^ Others have taken similar approaches to developing custom optimization routines that enable direct LETd optimization.^19^, ^20^, ^21^ Details of the developed systems communicate an intuitive experience similar to commercially available inverse optimization tools. The custom models and code complicate the methodology's implementation in other clinics. In addition to the direct optimization of biologically effective doses or LETd, indirect optimization of LETd based on track‐end penalties has been developed in research versions of clinical software.^22^ This again provides an intuitive, inverse optimization experience mimicking that of traditional planning only, with the addition of an objective in the optimization's cost function that penalizes protons stopping in normal tissue. Track‐end–based optimization's current use is limited to research software. Alternative, forward‐based planning strategies in beam setup have been explored and characterized in the literature.^23^ To summarize the state of biologically effective dose planning, the first two optimization strategies are not easily implemented by clinics‐at‐large, and the latter forward‐based strategies have been characterized in a limited way, often by comparing only a select strategy or two to each other.

Critically, we lack a set of tools and strategies that can be used without specially developed software or limited‐access research versions of treatment‐planning systems. Utilizing a previously published pipeline^24^ and Monte Carlo model,^25^, ^26^ we have been calculating LETd on selected clinical cases of concern. Briefly, this process utilizes our treatment‐planning system's application programming interface to dispatch treatment plans through a series of scripts that handle image preprocessing, job dispatch using TOPAS Monte Carlo code,^27^ post‐processing, and formatting of results into DICOM dose files. The pipeline removes user interaction with the command line, thus making it more accessible to all members of the clinical team. The use of Monte Carlo secondary calculations to evaluate both dose and LETd has become increasingly common in proton therapy. Details of alternative secondary systems have been published in the literature.^28^, ^29^, ^30^ We have approached our evaluation of LETd as an added dimension of uncertainty that we seek to minimize through up‐front treatment‐planning strategies. That is, how can we protect against the unknown effects of LETd, while preserving the dosimetric quality of the plan as traditionally judged with the use of a fixed RBE of 1.1?

The purpose of our study was to quantify the effect of treatment‐planning strategies that are readily available in current, commercially used treatment‐planning systems on LETd distributions. The comparisons were performed in a cylindrical water phantom to first characterize the effects independent of anatomic and other patient‐level variables. We then extended the planning strategies to four clinical cases to assess the impact in realistic clinical scenarios. The results provide guidance that is not dependent on in‐house software or Monte Carlo models.

## 2 MATERIALS AND METHODS

### 2.1 Planning conditions

The treatment‐planning strategies tested in this study were performed in two different scenarios: (1) a phantom‐based study and (2) planning on selected clinical cases. The first method was chosen to help control for interpatient variability that may affect the impact of a planning strategy or, potentially, exclude it due to an inability to achieve clinical dose constraints. The second provides clinical context in cases chosen as a representative sample of cranial irradiation for children with central nervous system (CNS) diagnoses. All planning strategies were used for the phantom‐based studies, but some were excluded for individual patients when clinically acceptable plans could not be achieved or were impractical given patient considerations.

#### 2.1.1 Phantom setup

A cylindrical water phantom of radius 8 cm and height 40 cm was oriented with its long axis along the superior/inferior direction. The orientation and radial symmetry of the phantom ensured that changes in coplanar beam angle would not be affected by oblique incidence on the phantom surface or entry through an edge created from a hexahedron‐shaped phantom such as a cube. The entire phantom volume was manually assigned a Hounsfield unit (HU) value of 0 in the treatment‐planning system.

**FIGURE 1:**
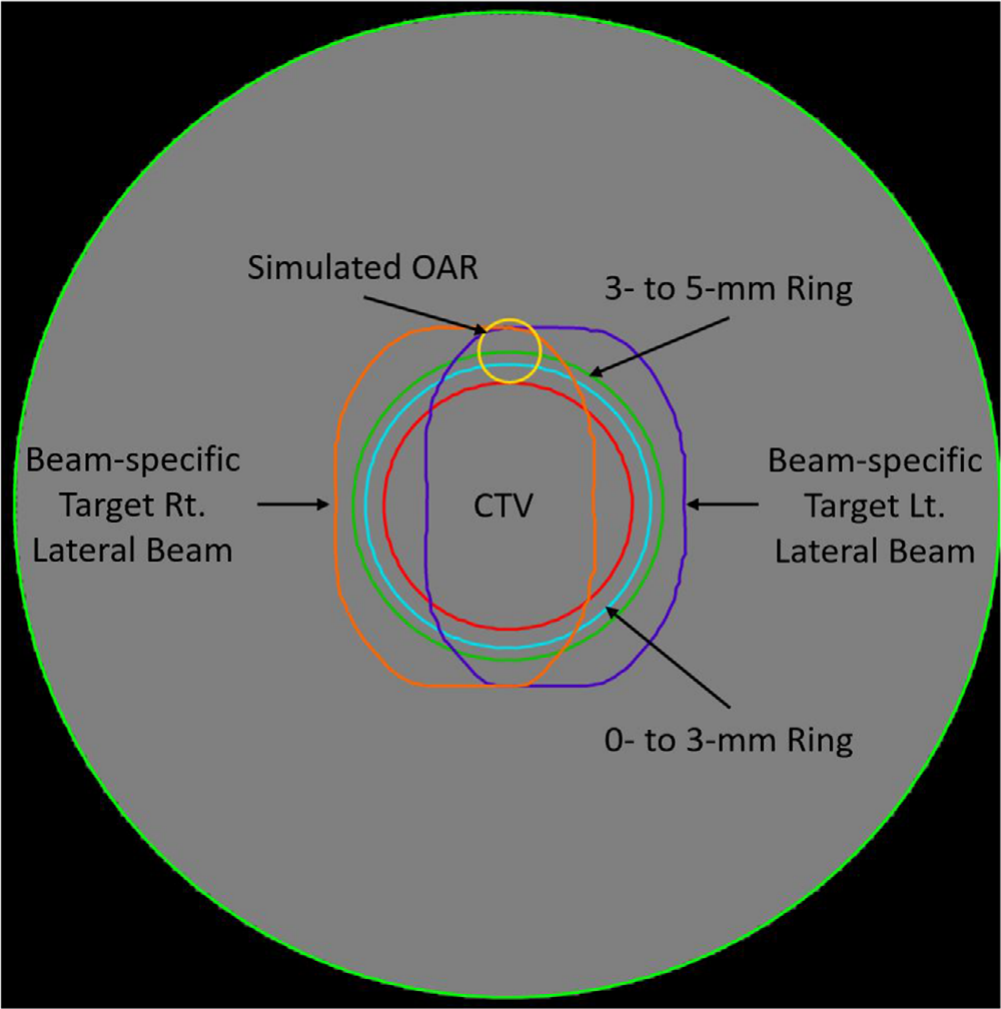
An axial slice of the cylindrical phantom is shown. The red contour is the cylindrical clinical target volume (CTV) created for planning. The cyan and green contours are concentric rings for evaluating dose‐weighted linear energy transfer immediately adjacent to the target. The orange and purple contours are beam‐specific target volumes (Section 2.2.6) created to prohibit spot placement along the distal edge of the CTV. The orange corresponds to spot placement for a beam entering on the left side of the image (patient right), and the purple, for a beam entering on the right side of the image (patient left). OAR, organ at risk; Rt, right; Lt, left

Within the cylindrical phantom, a centered, concentric cylindrical clinical target volume (CTV) of radius 2 cm and height 5 cm was defined (Figure 1). We created two concentric rings around the target volume, extending a radial distance of 0–3 and 3–5 mm from the surface of the target volume. Although the exact dose distribution needed to achieve robustness is case dependent, we can expect that the 0–3 mm ring will be largely covered when optimizing with robust scenarios of ±3%/3 mm. The ring from 3 to 5 mm is where we would expect to start to see dose falloff from the prescription dose. To probe the geometrical dependence of the LETd distribution that may be lost in radially symmetric regions of interest, a simulated organ at risk (OAR) was placed abutting the target volume.

#### 2.1.2 Patient cohort

Four patients were chosen to represent a diverse set of tumor shapes and locations within the brain: a 13‐year‐old girl with craniopharyngioma treated in the suprasellar region, a 9‐year‐old girl with medulloblastoma treated with a fourth‐ventricle boost following craniospinal irradiation, a 7‐year‐old boy with anaplastic ganglioglioma treated in the right frontal region, and a 14‐year‐old girl with a pilocytic astrocytoma treated in the region of the tectal plate. Axial and sagittal views of computed tomography images acquired for treatment planning are shown in the appendix (Appendix Figure A1) for each of the four patients.

### 2.2 Planning strategies

The baseline planning parameters are detailed in Table 1. In both phantom‐ and patient‐based studies, each test case differed from the baseline case in only one aspect of the planning strategy. All patients’ plans were robustly optimized using uncertainty scenarios of ±3%/3 mm. Our clinical practice is to cover the entire target volume with 95% of the prescribed dose (*V*_95%_) in the nominal treatment plan and cover 95% of the volume in all scenarios used during robust optimization. The dose coverage in nominal and uncertainty scenarios was prioritized in each treatment‐planning technique and case to focus the analysis on the difference in LETd distributions rather than any potential changes to the dose distribution. Each newly created treatment plan was designed by a single, experienced dosimetrist using the Eclipse treatment‐planning system v13.7 (Varian Medical Systems, Palo Alto, CA).

**TABLE 1:** Summary of the planning parameters for the baseline treatment plans for the water phantom and clinical patient studies

Treatment‐plan parameter	Water phantom	Patient 1	Patient 2	Patient 3	Patient 4
**Number of beams**	2 (opposed laterals)	2 (oblique laterals)	2 (oblique laterals)	2 (oblique laterals)	3 (oblique laterals and vertex)
**Intersection angle (**°)	180	150	170	140	N/A
**Optimization technique**	SFO	MFO	MFO	MFO	MFO
**Robust scenarios**	±3%/3 mm	±3%/3 mm	±3%/3 mm	±3%/3 mm	±3%/3 mm
**Use of RTVs**	No	No	No	Yes	Yes
**Nominal plan CTV—*V*_95%_**	100%	100%	99.4%	99.9%	99.8%

*Note*: The water‐phantom plan was created specifically for this study; the patient treatment plans were clinically delivered.

Abbreviations: CTV, clinical target volume; RTVs, robust target volumes.

The treatment‐planning strategies that were used and the parameters that were altered from baseline are described in detail in each of the subsequent sections.

#### 2.2.1 Number of treatment beams

**FIGURE 2:**
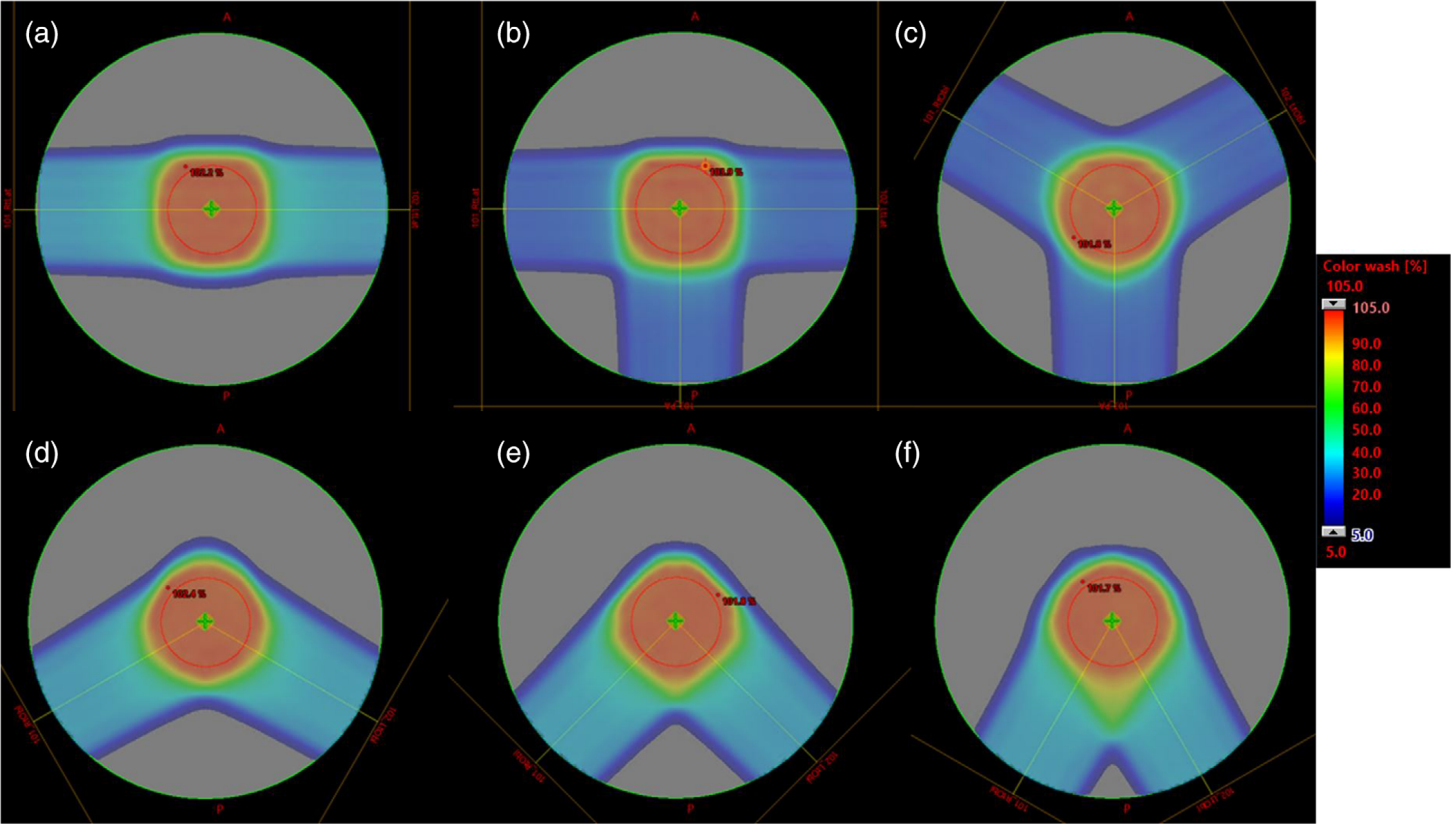
The five alternative beam orientations and the base plan, which utilizes opposed lateral fields. The alternatives to the base plan (a) are a three‐field orientation with opposed laterals and a single PA field (b); three coplanar, equally spaced beams (c); two beams with an intersection angle of 120° (d); two beams with an intersection angle of 90° (e); and two beams with an intersection angle of 60° (f)

We preferentially use two treatment fields for cranial proton radiation therapy. For this study, it is hypothesized that introducing a third field into the treatment will lead to the high‐LETd areas of each field being partially washed out by the lower LETd contributions from the remaining fields. In the water phantom study, we tested this by creating two treatment plans with three fields. One plan equally spaced each of the three coplanar beams so that the intersection angle between any two fields was 120°. We also created a three‐field plan in which two parallel opposed fields were supplemented with a third PA beam. This “T” orientation of the fields results in intersection angles of 90°, 90°, and 180° between the fields. The two plans utilizing three beams are shown in Figure 2, images (b) and (c).

When testing the addition of a third beam into the clinical plans, the geometry was not constrained to coplanar beams. Our only spatial constraint on the placement of the additional beam was to prohibit shooting through an OAR to achieve target coverage. As a result, we used an anteriorly angled vertex beam to treat a midline tumor (patient 1) and a PA beam to treat a midline tumor (patient 2) and posterior fossa tumor (patient 3). Patients 1 and 2 also had superiorly obliqued lateral beams. In the patient (patient 4) whose baseline plan included three beams, our modification involved reducing the number of beams to two.

#### 2.2.2 Intersection angle between beams

When possible, our clinical preference is to treat with parallel, or nearly parallel, opposed beams. The hypothesis is that the areas of high LETd at the distal edge of each beam start converging at the intersection as the intersection angle between beams decreases. In the extreme case of an intersection angle of 0°, the two fields would effectively become a single‐field plan in which the areas of high LETd are focused at the distal edge of the target, and no mitigation from an alternate beam is present to lower LETd.

In the phantom‐based studies, we successively moved the lateral beams into a symmetric and increasingly posteriorly obliqued pair. Starting with the parallel opposed beam, the intersection angle between beams was 180°, 120°, 90°, or 60°, respectively. The dose distributions for the plans are shown in Figure 2 images (a), (d)–(f).

The clinical implementation of this strategy was only used once for each patient, with beams obliqued at least 80°. Although this is unlikely to illustrate the extreme case, we thought the conservative change in angle provided a more clinically realistic beam orientation that aligned with what we see in our practice.

#### 2.2.3 Robust optimization scenarios

Two competing hypotheses could explain the effect of the scenario‐parameter magnitude in robust optimization. (1) As the scenario parameters increase (i.e., ±3%/3 to ±5%/3 mm), the volume of high LETd will increase as the surface area of the prescription dose expands to robustly cover the target. The increased volume will also occur at a distance further from the target volume, placing it closer to nearby critical structures. (2) The magnitude of high LETd is directly related to the steepness of the dose gradient in a plan, as a prior study demonstrating differences in LETd between pencil‐beam scanning proton therapy and passively scattered proton therapy suggested.^31^ As the magnitude of the scenarios is lowered, increasingly conformal doses and steeper dose gradients may be achieved. In both phantom‐ and patient‐based studies, the scenarios used for robust optimization were increased to ±5%/3 mm and decreased to ±2%/2 mm to assess the trade‐off in the hypotheses.

#### 2.2.4 Range shifter use

When using a nozzle‐mounted range shifter, we hypothesized that the increased spot size and increased spread in the energy spectrum at the point of entry into the patient would result in lower LETd values in the treatment plan. This strategy was tested only in the phantom‐based studies. Use in clinical plans would be highly dependent on the planning goals specific to the patient and associated planning constraints.

#### 2.2.5 Multifield and single‐field optimization

The treatment plans were optimized using both single‐field and multifield optimization strategies. Because multifield optimization enables greater dose heterogeneity on a beam‐specific level, we hypothesized that localized, beam‐driven hot spots would lead to an increase in the LETd. The clinical plans, whose target coverage was balanced against sparing nearby organs at risk, were most likely to exhibit this difference as the optimizer would push the multifield solution such that it was more likely to diverge from the single‐field optimization solution.

#### 2.2.6 Spot‐location restrictions and penalties

**FIGURE 3:**
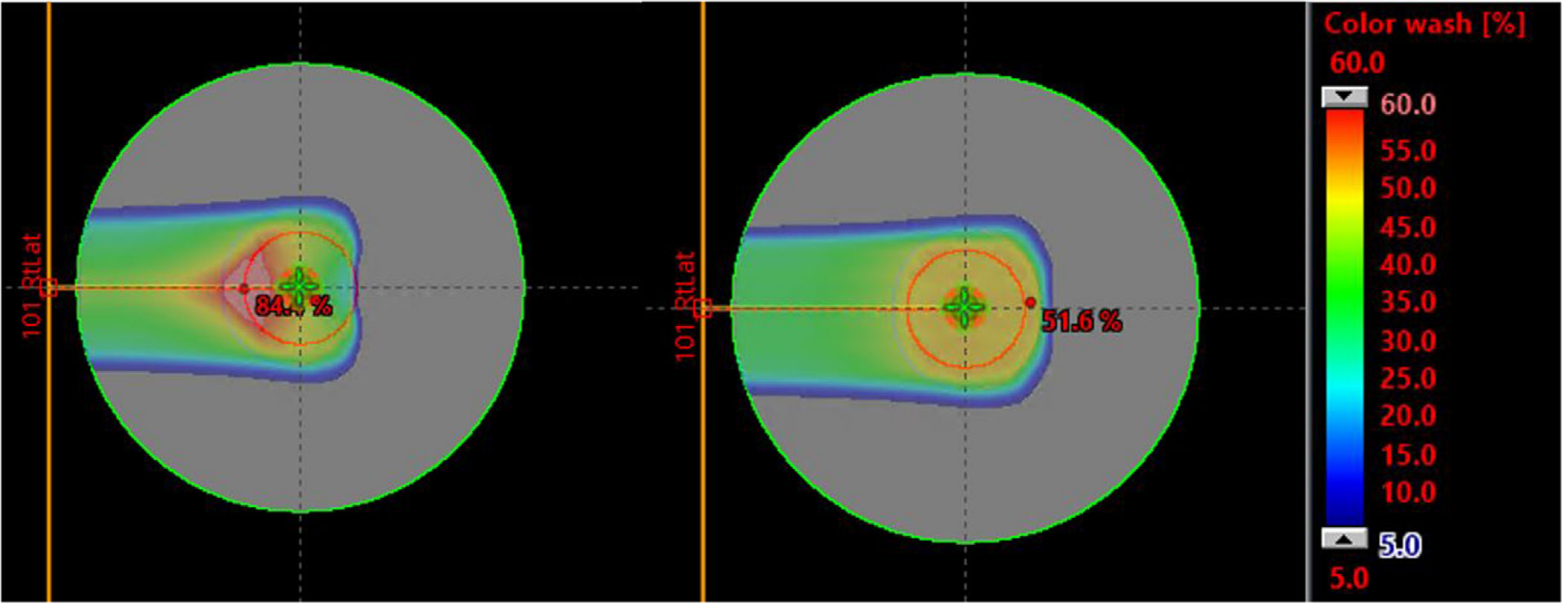
The dose contribution from a right lateral field in a parallel opposed plan is shown for the distal spot restricted planning (DSRP) approach (left) and the base plan (right) in the color wash image. The dose has been normalized to the prescription dose in this image. The DSRP plan prohibits spots along the distal edge of the target from each beam. The proximal portion of each beam is, therefore, forced to compensate for the opposing beam's lack of coverage. The base plan allows for uniform coverage from each of the opposed beams.

We created beam‐specific targets to restrict the placement of spots along the distal edge of a target or inside an OAR entirely. To do so, we used the treatment‐planning system's robust target volume (RTV) tool and labeled the technique as distal spot restricted planning (DSRP). The hypothesis was that the opposing beam would be forced to cover target volumes by preferentially weighting the proximal portion of the beam, resulting in reduced LETd outside the target volume. When implemented in the phantom‐based study, this meant prohibiting the beam from placing spots in the distal 5 mm of the target (Figure 3). In patient‐based planning, we started with the same 5‐mm reduction. In cases without parallel opposed beams, some amount of the CTV may be left uncovered after the 5‐mm reduction. In those cases, the beam‐specific targets were manually edited to ensure that the entirety of the CTV was covered by the union of all beam‐specific targets.

### 2.3 Monte Carlo calculations

All LETd calculations were performed by using the Monte Carlo multiparticle transport code Tool for Particle Simulations (TOPAS).^27^ Treatment plans were dispatched through a previously validated automated pipeline.^24^ The scored LETd came from the built‐in “Proton LET” tally in TOPAS. We transported primary and secondary protons as well as secondary neutrons and electrons during the simulations. The Proton LET scorer calculated LETd in consideration of primary and secondary protons, including energy deposited by secondary electrons. A total of 1.8 × 10^8^ histories were simulated per treatment field. As detailed in prior publications, we performed all simulations using the TOPAS Geant4 standard option 4 modules and default thresholds recommended by the Geant4 electromagnetic physics group. The final resolution of the LETd calculations matched the resolution of the CT grid, 0.47 × 0.47 × 1 and 0.98 × 0.98 × 1 mm^3^ for the phantom‐ and patient‐based studies, respectively.

### 2.4 Treatment‐plan analysis

All treatment plans were first evaluated by using a clinical, dose‐based assessment of plan quality to ensure that the alternative planning strategies did not produce unrealistic treatment plans. Emphasis was placed on covering the CTV with 95% of the prescription dose and minimizing a hot spot surrogate ($D_{0.03\;cm^{3}}$) in the patient. Table 2 summarizes both the physical dose and LETd metrics used in plan evaluation.

**TABLE 2:** Summary of evaluation metrics used to assess plan quality for dose and biological surrogates

Plan evaluation metric	Description
CTV *V*_95%_ (% of structure volume)	Volume of the CTV that receives a dose of at least 95% of the prescription dose
CTV *D*_min_ (% of Rx dose)	Minimum dose within the CTV
$D_{0.03\;cm^{3}}$ (% of Rx dose)	Minimum dose to the hottest 0.03 cm^3^ (hot spot surrogate)
Mean LETd (keV/μm)	Mean LETd in specified structure
Max LETd (keV/μm)	Maximum dose‐weighted linear energy transfer in specified structure

*Note*: Relative doses are reported in relation to the prescription (Rx) dose.

Abbreviations: CTV, clinical target volume; LETd, dose‐weighted linear energy transfer.

The mean LETd was evaluated in each of the concentric rings around the target. The concentric rings enable the comparison of the LETd distribution in normal tissues immediately adjacent to the target to that inside the target, where the increased LETd may be clinically beneficial. Various planning techniques might result in similar integral LETd values having different spatial distributions. To assess the spatial dependence, the LETd distribution within the adjacent simulated OAR was evaluated.

## 3 RESULTS

### 3.1 Water phantom simulations

Among the 11 tested planning scenarios, 10 had comparable dosimetric quality. The DSRP approach, in which allowable spot positions are cropped from the distal edge of the target, resulted in reduced coverage (*V*_95%_ = 99.6%) and increased maximum dose ($D_{0.03\;cm^{3}}$ = 107.6% of the prescription dose). All other planning techniques could cover the CTV with at least 95% of the prescription dose.

The mean and maximum LETd in the CTV and concentric rings for each of the plans are presented in Table 3. Increased LETd within the target volume occurred with the DSRP approach, whereas decreased LETd occurred when using a range shifter or adjusting the robustness parameters in scenario‐based optimization. In the concentric rings outside the target, the largest increase in LETd compared to the base plan occurred as the intersection angle between incident beams was reduced. Maximum LETd in the surrounding 0 to 3 mm was 10.2, 10.0, 8.2, and 7.8 keV/μm for 60°, 90°, 120°, and 180° (base), respectively. Although the LETd in the 0–3‐mm ring surrounding the CTV was reduced when the number of beams changed from 2 to 3, this was true only when the beams were evenly spaced. Two opposed beams with a posterior beam, as is often used clinically, resulted in LETd comparable to the base plan in both rings (0–3 and 3–5 mm) surrounding the CTV. The DSRP technique, an approach intended to reduce LETd immediately outside the target, resulted in the largest increase in maximum LETd both inside and outside the CTV.

**TABLE 3:** A summary of the mean and maximum dose‐weighted linear energy transfer (LET) values in the simulated clinical target volume (CTV), a ring extending 0–3 mm around the CTV, and a ring extending 3–5 mm around the CTV

	LETd (keV/μm)					
	CTV		0–3 mm ring		3–5 mm ring	
Planning strategy	Mean	Max	Mean	Max	Mean	Max
**Base plan**	4.5	6.2	5.3	7.8	5.7	7.9
**3 beams—even spacing**	4.6	6.5	5.4	6.8	5.7	7.1
**3 beams—T shape**	4.6	6.8	5.4	7.5	5.7	7.9
**Intersection angle—60°**	4.5	7.4	5.3	10.2	5.8	12.2
**Intersection angle—90°**	4.6	7.1	5.5	10.0	**6.0**	10.9
**Intersection angle—120°**	4.5	6.4	5.3	8.2	5.7	8.9
**MFO**	4.7	6.2	5.4	7.7	5.6	7.7
**Distal spot restricted planning**	**6.0**	**13.9**	**5.8**	**14.6**	5.2	**14.5**
**Range shifter**	3.9	5.9	5.1	7.7	5.4	7.8
**RTV**	3.9	6.1	5.1	7.5	5.3	7.4
**Robust scenario 2%/2** **mm**	3.9	7.0	5.5	7.5	5.6	7.3
**Robust scenario 5%/3** **mm**	3.9	5.7	5.0	7.5	5.5	8.1

*Note*: Underlined and bolded values indicate the largest magnitude in each column.

Abbreviation: LETd, dose‐weighted linear energy transfer RTV, robust target volume.

**FIGURE 4:**
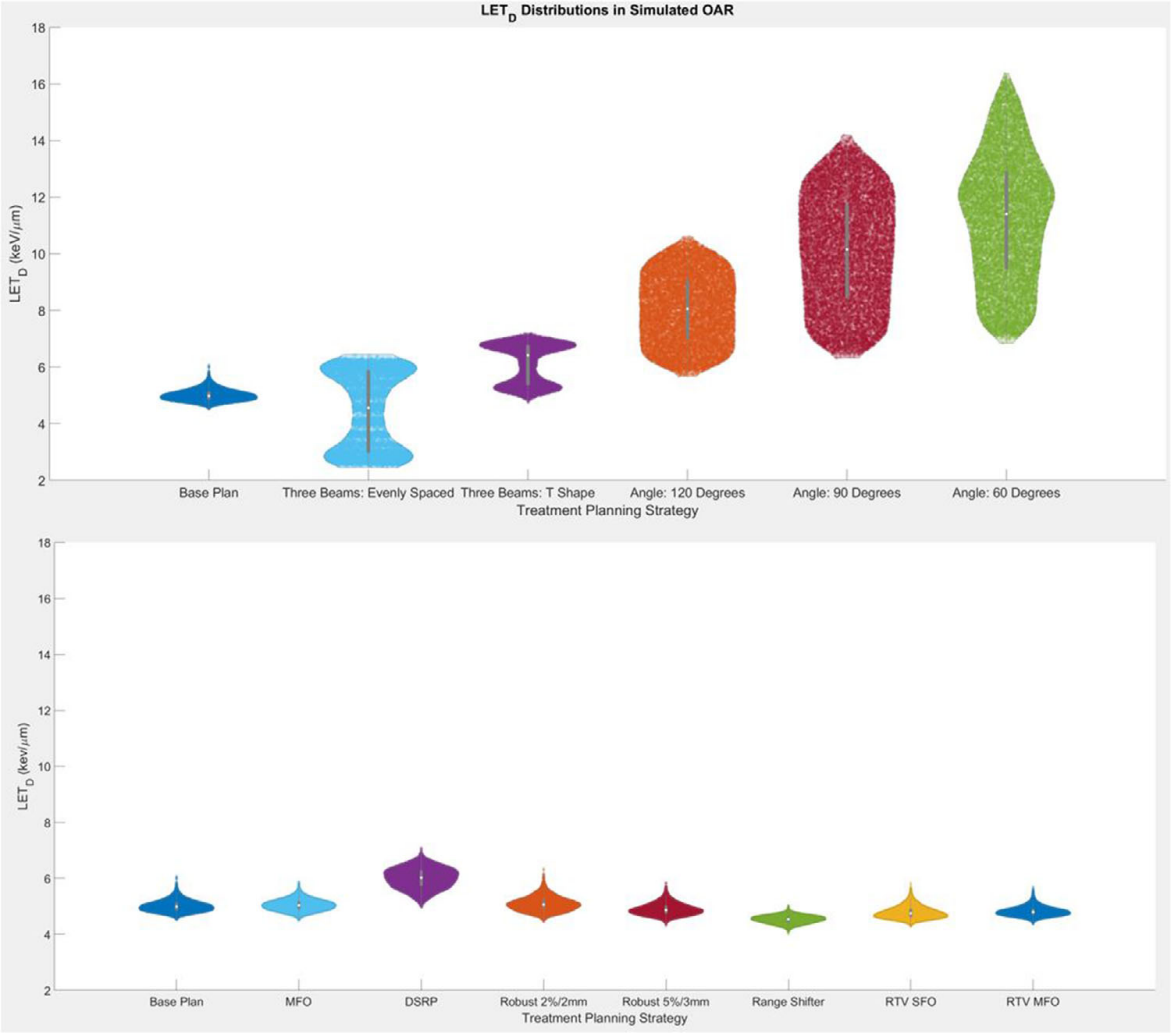
A violin plot of the dose‐weighted linear energy transfer (LETd) distribution within the simulated organ at risk in a phantom‐based study. The width of the plots corresponds to the frequency of occurrence in LETd values at the corresponding location on the *y*‐axis (LETd). Each of the treatment‐planning techniques is shown as an individual violin plot in the figure. Comparisons of treatment beam configuration are displayed on the top and comparisons of all other plans are displayed on the bottom.

The need to consider the spatial distribution of LETd is most evident in evaluating the simulated OAR for each of the planning strategies. Violin plots in Figure 4 show the distribution of LETd values in the simulated OAR. A clear trend toward higher LETd (mean and maximum) is observed as the intersection angle is reduced. Both three‐beam plans show maximum LETd values (6.4 and 7.2 keV/μm for evenly spaced and T‐shaped, respectively) higher than those of the base plan (6.1 keV/µm). Interestingly, the mean LETd in the evenly spaced three‐beam plan (4.5 keV/μm) was lower than that of the base plan (5.0 keV/μm). Comparing the distribution of the violins in the two cases demonstrates that the mean may be a deceiving metric given the bimodal distribution of the evenly spaced three‐beam plan. The DSRP results exhibited an overall increase in LETd in the simulated OAR. The remaining planning techniques had minimal impact on the deviations in LETd from the base plan. Within the simulated OAR, $D_{0.03\;cm^{3}}$ was within ±1% of the prescription dose with respect to the base plan for all but one planning technique: the reduced scenario parameters used in the ±2%/2 mm plan. For the reduced scenario‐parameters plan, $D_{0.03\;cm^{3}}$ was reduced from that of the base plan by 4 percentage points.

### 3.2 Clinical treatment plans

Of the four patients selected for alternative treatment‐planning strategies, dosimetric quality similar to that of the original clinical plan was achieved in three. Data from patient 4, whose plan was clinically unacceptable, was removed from LETd analysis. The resulting plans for this patient failed to achieve target coverage that we would have required for clinical treatment in our practice, and we reasoned that any LETd comparisons would be unrepresentative of clinical evaluations.

**FIGURE 5:**
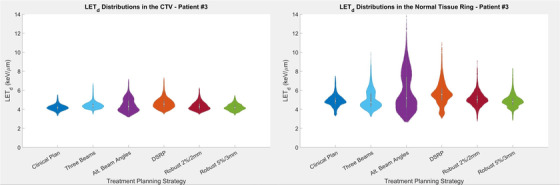
A series of violin plots demonstrating the distribution of dose‐weighted linear energy transfer (LETd) in the clinical target volume (CTV) (left) and normal‐tissue ring (right) defined as the area between a 3 and 5‐mm expansion of the CTV. Results are presented for patient 3. The central dot in each plot is the mean of the distribution, and the centered bars represent one standard deviation.

Figure 5 shows the variation in LETd distributions among planning techniques in a series of violin plots for patient 3 in both the CTV and normal‐tissue ring, defined as the area between a 3‐mm and 5‐mm expansion of the CTV. Corresponding plots for patient 1 (Appendix Figure A2) and patient 2 (Appendix Figure A3) are included in appendix. Although differences in mean LETd up to 1.0 keV/μm occurred in the surrounding normal‐tissue ring (patient 3, alternate beam angles plan), no consistent trends occurred across patients or planning strategies in the CTV or normal‐tissue ring. Observed differences within the target volume were less than the surrounding normal tissue changes, with the maximum difference in mean LETd compared to the base clinical plan being 0.4 keV/μm (patient 3, distal spot restricted plan).

**FIGURE 6:**
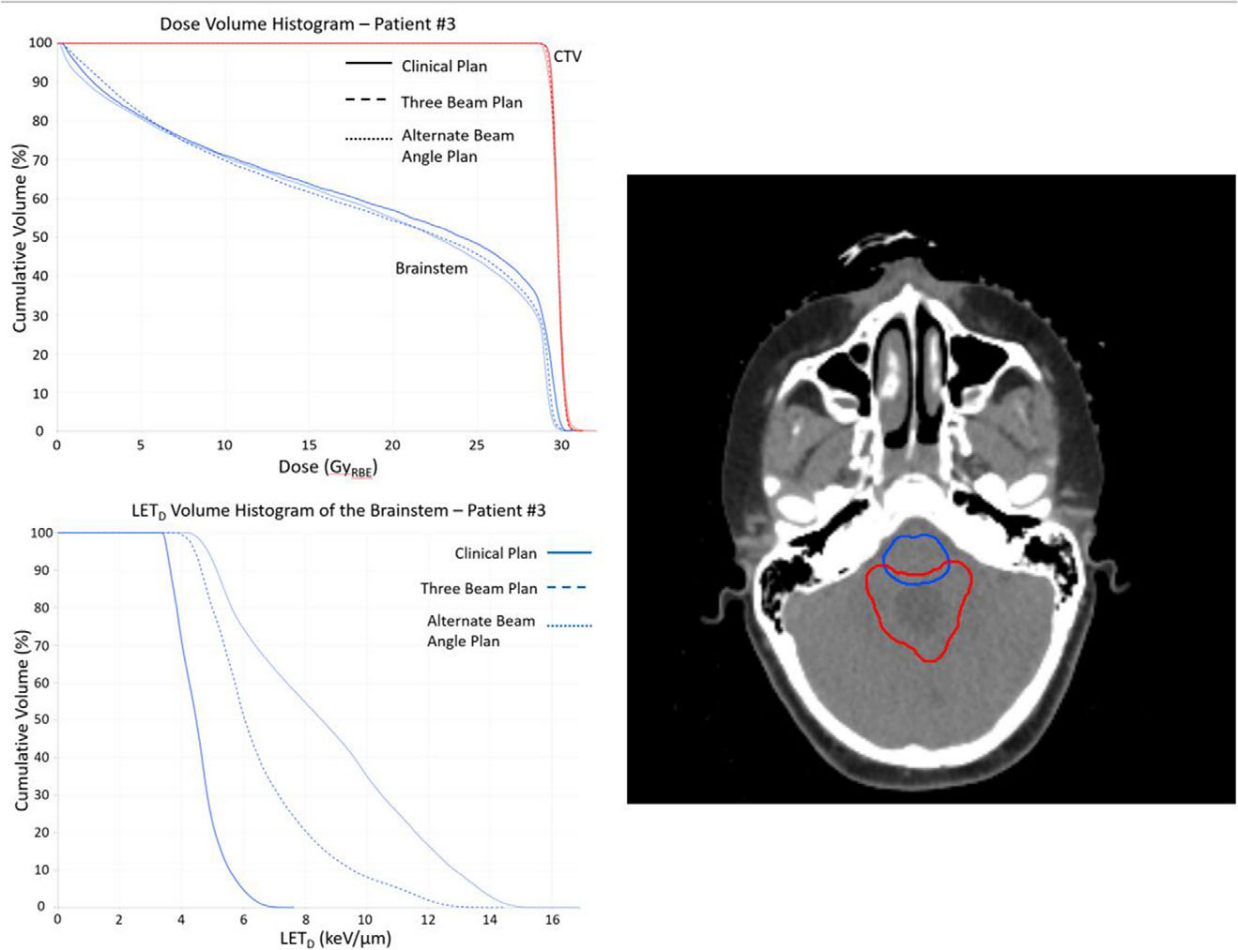
A dose–volume histogram (top left) and dose‐weighted linear energy transfer (LETd)–volume histogram (bottom left) are shown for patient 3. An axial image (right) showing the clinical target volume (CTV) (red) and brainstem (blue) is shown for reference. The prescription dose for the treatment plans generated was 30.6 Gy_RBE_.

To evaluate whether the treatment beam geometries affected a patient case as they did the phantom, we evaluated the LETd in the brainstem of patient 3. The target, located in the posterior fossa, and immediately adjacent brainstem, located anterior to the target, had geometries similar to the phantom's geometry. The posterior fossa is the most common site of occurrence in pediatric CNS tumors,^32^ so the case is representative of a common clinical scenario. Figure 6 shows the dose–volume histogram (DVH) and LETd–volume histogram (LVH) for the clinical plan (two beams, 140° angle between beams), three‐beam plan, and a treatment plan with a beam intersection angle (80°) smaller than that of the clinical plan. A similar trend to that observed in the phantom is present, with the three‐beam plan causing an increase in LETd in the brainstem and the smaller beam intersection angle having the largest impact on the LETd. As seen in the DVH, all three plans produced similar dose distributions in the CTV and brainstem despite the dramatic difference in LETd observed in the brainstem.

## 4 DISCUSSION

In this study, we quantified the effect of forward‐based treatment‐planning strategies on LETd distributions. We took a systematic approach in which we first evaluated different planning strategies in a cylindrical water phantom. The water phantom comparisons allowed for a direct comparison of treatment‐planning techniques without the need to consider how patient anatomy may affect the suitability of each planning technique. Following the phantom analysis, we replanned four clinically used patient treatment plans using the different treatment‐planning strategies. Three of the four patients received dosimetrically acceptable treatment plans, enabling an evaluation of the strategies in a clinically relevant manner. The failure of treatment plans in one of the four patients to reach acceptability suggests that alternative treatment‐planning strategies are largely possible but not universally applicable. In both water phantom and patient evaluations of LETd distributions, results point toward LETd being strongly dependent on treatment‐planning geometry, which is the choice of number of beams, beam angles, and relationship with nearby organs at risk.

Our study's strength lies in the broad range of planning strategies compared within a single planning study. All planning strategies are forward‐based and accessible by using available tools in current clinical treatment‐planning systems. The phantom‐based comparisons provide the most sterile comparison of the effect of plan technique on LETd. When translated to clinical cases of varying anatomical location, the impact becomes less clear. Therein lies the biggest weakness of the study: Our number of clinical cases was limited and variable enough to make drawing clinical conclusions challenging. We postulate that the breadth of planning techniques implemented by a board‐certified dosimetrist provides an adequate survey to at least comment on the techniques’ applicability in clinical cases. Ideally, a much larger sample size would be used. We maintain that replanning the same patients with all techniques was more valuable than comparing strictly clinical plans selected to represent the various planning strategies. Looking exclusively at clinically treated plans is likely to yield heavily biased results due to case considerations that push the planning strategy toward a specific solution. With at least six additional planning strategies being used per patient, an adequate sample size for strong conclusions becomes prohibitively time‐consuming unless plan quality and clinical realism are sacrificed.

The AAPM's recent report on the RBE of proton therapy acknowledges that the uncertainties in RBE as a function of LETd are too high to be implemented within an acceptable error margin.^4^ Our study avoided comparing treatment‐planning techniques using different RBE models for this reason. What is consistent among the models is that an increase in LETd leads to an increase in the biological effectiveness of a comparable physical dose. Our analysis, interpretation of the data, and commentary on treatment‐planning strategies are strengthened by their consistency with the report by way of respecting the correctly highlighted uncertainties and not relying on any one RBE model being correct or “best.”

The phantom‐based comparisons provided the best controlled comparison of planning strategies. There were clear trends toward increasing LETd in both normal‐tissue rings surrounding the target when decreasing the intersection angle between treatment beams and adding a third, posterior beam to the parallel opposed beams. Decreasing the intersection angle and adding a third posterior beam are strategies driven by geometric considerations that produce more intuitive changes in LETd when considering the distal region of the beam path. In our clinical practice, we first prioritize these types of considerations in cases in which surrounding normal tissue may be especially radiosensitive or of critical functional importance.

The work published by Giantsoudi et al.^23^ provides the best comparison for our observations when evaluating two versus three beams in treatment plans. Differences between their study and ours are their use of passively scattered proton therapy, a known contributor to differences in LETd,^31^ a fixed‐range uncertainty of 3.5%/1 mm rather than scenario‐based robust optimization, and their use of a strategy shooting through the brainstem entirely to avoid track ends within the structure. Their three‐field, dose‐sparing technique (analogous to our three‐beam arrangement with two laterals and a single PA field) showed an increase in mean LETd of 1.0‐keV/μm anterior to the target more than the two‐field plans. Although our phantom study comparison did not show an appreciable change in mean LETd in the surrounding 0–3‐mm ring by adding a posterior beam to the opposed lateral plan (+0.1 and −0.3 keV/μm for mean and maximum, respectively), if we consider a simulated OAR immediately anterior to the mock target volume, a difference of +1.1 keV/μm was observed in both the maximum and mean LETd. With $D_{0.03\;cm^{3}}$ being within ±1 percentage point between techniques, we feel it is reasonable to conclude that these differences are not due to inherently different dose distributions. The anatomy of patient 3 in our study closely resembles the clinical anatomy of their study, which was also an examination of posterior fossa tumors. Our results in Figure 6 show an increase in maximum LETd of 6.8 keV/μm and an increase in the mean LETd of 2.1 keV/μm. Our phantom study results demonstrate results similar to theirs. Our patient example matching their study showed a more dramatic effect in LETd changes with a third beam, perhaps due to the previously mentioned differences in delivery technique. The comparison of these specific geometries is the motivation behind our preferred use of two opposed lateral beams when possible.

The use of beam‐specific RTVs is a strategy normally used to improve a plan's dosimetric robustness. In the phantom comparison, the mean LETd in the CTV was lower when using RTVs (3.9 keV/μm) than it was in the base plan (4.5 keV/μm). We attributed the reduction in LETd to a more efficient distribution of allowable spot locations. The improved dosimetric robustness with RTVs is most obvious in areas of heterogenous tissue along the beam path. We hypothesize that the change in LETd would be most obvious in these situations as well. It is a difficult hypothesis to prove in the limited set of clinical plans that we examined and is likely to be highly patient‐specific.

Changing the optimization from single‐field to multifield did not noticeably affect the mean and maximum LETd values in the regions evaluated. Due to the simple geometry in the phantom‐based study, the optimizer's solutions in the two optimization schemes should be similar. The difference would likely be more obvious in plans that require heavy modulation to spare nearby critical structures. This, unfortunately, did not present as obvious in the comparisons of clinical treatment plans.

Using a range shifter had the most measurable impact on LETd values within the CTV (mean LETd 0.6 keV/μm less than that of base plan) and minor effects in the surrounding 3–5‐mm ring (mean and maximum LETd 0.2 keV/μm less than that of base plan). The more favorable LETd distribution comes at the expense of increasing spot size, and by extension, the penumbra and dose sparing achieved in the plan. Both effects should be noted to also improve robustness. Therefore, the strategy should be carefully evaluated against the need to achieve highly conformal doses and scenario‐based dose distributions.

Our approach of using beam‐specific targets to prohibit spot placement along the distal portion of the target (DSRP) resulted in the largest LETd among the techniques (increase of 1.5 keV/μm in the mean LETd in the CTV and 6.6 keV/μm in the maximum LETd in the distal normal‐tissue ring with respect to baseline plan). Compared to the reductions of mean and near‐maximum LETd of at least 2 keV/μm in the work presented by Traneus et al.,^22^ our results were disappointing. We attributed the difference in results to their implementation in which track ends were penalized in the objective function of the optimization rather than outright prohibited as in our DSRP approach. The hot streaks in dose and LETd observed in our phantom‐based plans—and to a lesser extent, clinical plans—suggested that the optimizer was competing against itself in the scenario‐based optimization used for robustness. With regions of target and surrounding tissue needed for robust coverage being restricted in spot placement, high doses were needed to achieve coverage in the robust scenarios. It is possible that more carefully splitting the target for each beam with the chosen robust scenarios in mind could result in something closer to our intended results. This may come at the expense of increased proximal dose and would become highly specific to the clinical case and robust parameters used during optimization. Despite results not meeting our expectations, we felt that they provide an important lesson in not trying to “trick” the optimizer into a spot pattern and weighting that might be more favorable. At the very least, institutions should be careful in evaluating if the final effect matches the intended outcome. Additionally, our comparison of LETd in DSRP to standard optimization points strongly toward objective‐based strategies as proposed in the Traneus study rather than forward‐based spot limitations set prior to optimization.

**FIGURE A4:**
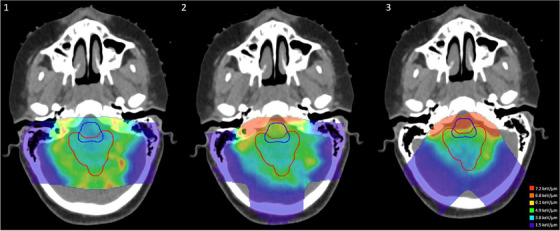
Axial computed tomography images with dose‐weighted linear energy transfer overlaid are shown for patient #3. The base, clinical distribution (#1, left), a three‐beam orientation (#2, center), and the alternate beam angle setup (#3, right) are shown as representative distributions for the respective planning strategies. The clinical target volume is delineated in red and the brainstem in blue.

Although mean LETd and high‐risk volumes did not show systematic differences across patients in the CTV and normal‐tissue ring, trends were observed in the location of areas of high LETd, corresponding to variations in the beam angles and number of beams used. The example of patient 3 in Figure 5 shows the clearest example of the trend. The baseline clinical plan was two opposed lateral beams treating a fourth‐ventricle volume after craniospinal irradiation. The three‐beam plan resulted in a 0.2 and 0.1 keV/μm increase in the mean LETd within the CTV and 3–5 mm surrounding tissue, respectively. Although quantitatively small, the shift and concentration of the higher LETd to an area adjacent to and overlapping the brainstem would be of clinical concern. The alternative‐beam‐angles plan, in which the intersection angle between beams was reduced, shows the largest observed difference in mean LETd in the 3–5 mm normal‐tissue ring (0.9 keV/μm) from that of the base plan. Although the plans produced similar dosimetric results and exhibited minimal changes in LETd in the CTV, the differences observed in the brainstem were dramatic. The LETd distributions in the three plans are shown in the axial plane in Figure A4. The plans in Figure 6 are consistent with our experience in which treatment and anatomic geometry—that is, beam approach with respect to surrounding normal tissue—are more predictive of undesirable LETd distributions in areas of high dose.

Our study was motivated in part by the uncertainties surrounding various biological models that seek to establish a relationship between RBE and LETd. As a result, we avoided the use of RBE models in our analysis and focused on LETd as a surrogate that might inform us on just how robust we are with respect to biologic uncertainties. The use of RBE models can still be helpful in contextualizing the magnitude of LETd changes within our study. The model proposed by McNamara et al.^12^ is most helpful in evaluating the RBE changes in the CTV as it allows for non‐OAR alpha/beta ratios to be considered. The maximum difference in mean LETd in the CTV that we observed in the phantom study was from 4.5 keV/μm in the base plan to 3.9 keV/μm when using a range shift or altering robust scenarios. Assuming an alpha/beta ratio of 10 Gy that changes in LETd corresponds to an RBE change from 1.13 to 1.11. An examination of results in an OAR where an alpha/beta ratio of 3 Gy may be assumed makes the results more striking. The McNamara model estimates that the increase in mean LETd of 2.1 keV/μm observed in patient 3 corresponds an increase in the RBE from 1.26 to 1.35, or a 7% relative increase in biologic dose. A similar model proposed by Tseung et al.^18^ estimates that the same increase in LETd corresponds to an increase in RBE from 1.29 to 1.41. In both models, the lower alpha/beta ratio of an OAR combined with higher LETd values results in increased baseline RBE values and more pronounced changes in RBE resulting from different planning techniques.

## 5 CONCLUSION

Our study provides a survey of forward‐based planning strategies available to clinical teams without purchasing or developing specialized systems capable of biological treatment planning in proton therapy. The most consistent results were observed with planning strategies relating to treatment beam geometry with respect to anatomic location of the target. That is, avoiding beams that stop immediately proximal to critical structures and utilizing large intersection angles between treatment beams is the surest way to mitigate the potential impact of biologic uncertainties associated with high LETd. Given our study and clinical experience, we recommend using two parallel opposed beams in midline, cranial tumors when possible. Range shifter use intended to influence LETd and alternative strategies of spot‐placement restrictions should be used sparingly and only when clinics can calculate the resulting LETd to evaluate against the alternate planning strategies for the specific case. Even without accepted biological models in place, there remains a need for commercially available, inverse‐planning tools to either directly optimize LETd or proton track ends to arrive at more biologically robust treatment plans without sacrificing dosimetric plan quality.

## AUTHOR CONTRIBUTION

Each of the others has contributed to the conception, design, acquisition, analysis, and reporting of the work contained in this manuscript.

## CONFLICT OF INTEREST

The authors have no relevant conflicts of interest to disclose.

## Figures and Tables

**FIGURE A1:**
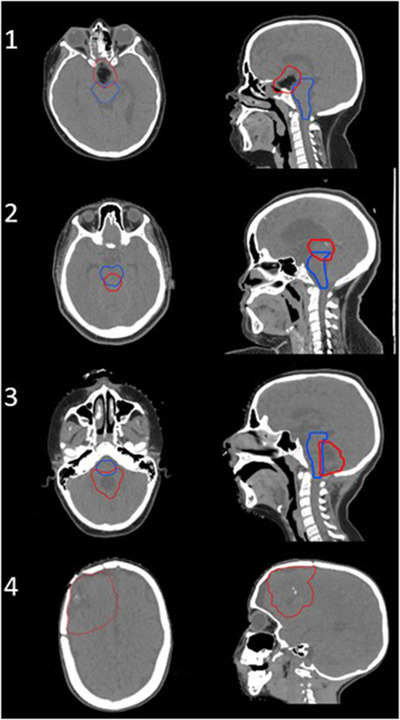
Axial (left) and sagittal (right) computed tomography images for each of the four patients used in the clinical planning study. The clinical target volume (CTV) is delineated in red. When the CTV was near or overlapping the brainstem (blue), that contour is also shown.

**FIGURE A2:**
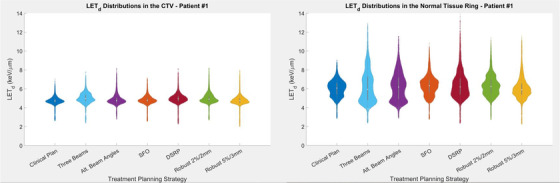
A series of violin plots demonstrating the distribution of dose‐weighted linear energy transfer (LETd) in the clinical target volume (CTV) (left) and normal‐tissue ring (right) defined as the area between a 3 and 5‐mm expansion of the CTV. Results are presented for patient 1. The central dot in each plot is the mean of the distribution, and the centered bars represent one standard deviation.

**FIGURE A3:**
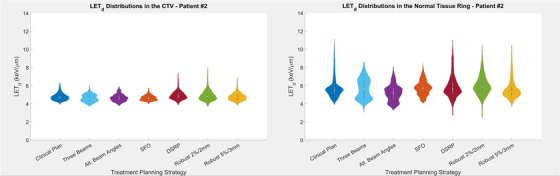
A series of violin plots demonstrating the distribution of dose‐weighted linear energy transfer (LETd) in the clinical target volume (CTV) (left) and normal‐tissue ring (right) defined as the area between a 3 and 5‐mm expansion of the CTV. Results are presented for patient 2. The central dot in each plot is the mean of the distribution, and the centered bars represent one standard deviation.

## 1

Figure A1, A2, A3, A4
